# Association between Lipoprotein Subfractions, Hemostatic Potentials, and Coronary Atherosclerosis

**DOI:** 10.1155/2022/2993309

**Published:** 2022-08-30

**Authors:** Tadeja Poropat Flerin, Mojca Božič Mijovski, Borut Jug

**Affiliations:** ^1^Institute of Radiology, University Medical Centre Ljubljana, Zaloška cesta 7/I, SI-1000 Ljubljana, Slovenia; ^2^Department of Vascular Diseases, University Medical Centre Ljubljana, Zaloška cesta 7/VI, SI-1000 Ljubljana, Slovenia; ^3^Faculty of Pharmacy, University of Ljubljana, Aškerčeva 7, SI-1000 Ljubljana, Slovenia; ^4^Faculty of Medicine, University of Ljubljana, Vrazov trg 2, SI-1000 Ljubljana, Slovenia

## Abstract

**Background:**

Dyslipidemias are associated with atherosclerotic plaque formation and a prothrombotic state, thus increasing the risk of both atherosclerotic vascular disease and atherothrombotic adverse events. We sought to explore the association between lipoprotein subfractions, overall hemostasis, and coronary calcifications in individuals at intermediate cardiovascular risk.

**Methods:**

Consecutive statin-naive individuals at intermediate cardiovascular risk referred for coronary artery calcium score (CACS) scanning were included. CACS was assessed using a 128-slice dual-source CT scanner. Traditional lipid profile, high-density lipoprotein (HDL) subfractions 2 and 3, and small dense low-density lipoproteins (sdLDL) were measured with commercially available assays. Overall hemostatic (OHP) and coagulation potentials (OCP) were measured spectrophotometrically, using fibrin aggregation curves after exposure to thrombin and recombinant tissue-type plasminogen activator, respectively. Overall fibrinolytic potential (OFP) was calculated as a difference between the two areas under curves.

**Results:**

We included 160 patients (median age 63 (interquartile range (IQR), 56-71 years, 52% women, and median CACS 8, IQR 0-173 Agatston units). HDL3 levels—but not sdLDL or hemostatic potentials—were significantly associated with CACS zero, even after adjusting for age, sex, arterial hypertension, dyslipidemia, diabetes, and smoking history (OR 0.980 (0.962-0.999), *p* = 0.034). HDL3 was also significantly associated with OCP (*r* = −0.232, *p* adjusted for age and sex 0.037).

**Conclusions:**

In patients at intermediate cardiovascular risk, HDL3 is associated with both subclinical atherosclerosis and overall coagulation. Our findings are in line with studies reporting on an inverse relationship between HDL3 and atherosclerosis and provide one possible mechanistic explanation for the association between novel lipid biomarkers and coagulation derangements.

## 1. Introduction

Atherosclerotic vascular disease is characterized by progressive vascular involvement possibly resulting in atherothrombotic events, such as myocardial infarction or stroke. On the one hand, dyslipidemia is an established driver of atherosclerotic vascular disease occurrence and progression [1]; on the other hand, dyslipidemia may be associated with a prothrombotic state and therefore represents the link between atherosclerotic vascular disease and its progression to atherothrombotic events [2].

Traditional lipid derangements—i.e., especially high low-density lipoprotein cholesterol (LDL-C) levels—have been identified as the most important independent marker of high atherosclerotic vascular risk [1, 3]. However, despite aggressive LDL-C lowering, substantial residual atherosclerotic risk remains and may possibly be associated with lipid metabolism derangements not captured by measuring LDL-C [4]. For one, the inverse relationship between high-density lipoprotein cholesterol (HDL-C) and cardiovascular risk is well documented; however, the relationship is not as consistent as for LDL-C, and interventions lowering HDL-C have indeed failed to prove a definite causal relationship [5, 6]. The association between HDL-C and cardiovascular risk may possibly be confounded by the role of different HDL subtypes in atherosclerotic disease pathophysiology. HDL is a complex lipoprotein, with over 100 structural proteins possibly affecting its function. Subfractioning to large, lipid-rich HDL2 and small, protein-rich HDL3 (reflecting lipoprotein maturation through reverse cholesterol transfer pathway) may provide a better appreciation of the role of HDL in cardiovascular pathophysiology as compared to standard measuring HDL concentration [7]. Yet, epidemiological studies have been inconclusive—some studies suggested antiatherogenic potential of HDL2 as opposed to HDL3, while others do not [8].

Lipid derangements may also promote a procoagulant state, which is paramount for the progression of atherosclerosis to thrombotic artery occlusion [9]. Such a procoagulant state is usually not detected by screening coagulation tests (i.e., prothrombin time (PT) and activated partial thromboplastin time (aPTT)), whereas steady-state fibrinogen levels are difficult to determine because of very high intraindividual biological variation [10]. Previous studies have predominantly reported on the association between LDL-cholesterol and individual coagulation factors, such as Factor VIII, von Willebrand factor, antithrombin, and protein C [11–13], while the association with other and/or advanced lipid parameters, such as small dense LDL (sdLDL) and HDL subtypes, has not been as thoroughly addressed. There is some evidence that HDL3—but not HDL2—may negatively modulate fibrinolysis *in vivo* through increased levels of plasminogen activator inhibitor-1 (PAI-1) secreted from adipocytes and therefore could be associated with atherothrombosis [14]. Other studies, conversely, demonstrate the HDL3 mediated the inhibition of thrombin-induced fibrinogen binding and platelet aggregation, thus implying its antithrombotic properties [15].

Individual hemostatic factors are also independently associated with the presence, severity, and prognosis of coronary artery disease [16, 17]; the association, however, is modest at best and often counterintuitive, which likely reflects the involvement of individual factors in complex activation-inhibition hemostatic pathways [18]. Contemporary assessment of hemostasis is therefore shifting towards global assays, such as *in vitro* thrombus formation, which may better capture the coagulation-versus-fibrinolysis equilibrium and translate the complexity of hemostatic derangements into a single overall measure. Global hemostatic, coagulation, and fibrinolytic potentials (OHP, OCP, and OFP) represent such measure—by quantifying the rate of *in vitro* fibrin formation (i.e., adding thrombin for coagulation appraisal) and fibrin degradation (i.e., adding tissue plasminogen activator for fibrinolysis appraisal) [19, 20]. OHP, OCP, and OFP have been validated in healthy individuals and different groups of patients, including in patients with coronary artery disease [21, 22].

Hence, in the present study, we aimed to find associations between advanced lipid parameters, global hemostatic assays, and atherosclerotic vascular disease. We hypothesised that lipid subtypes (i.e., sdLDL and HDL3/HDL2), overall hemostatic potentials (i.e., OHP, OCP, and OFP), and coronary atherosclerosis (as determined by the coronary calcium score (CACS)) would be correlated in individuals at intermediate cardiovascular risk.

## 2. Methods

This was a single-centre cross-sectional cohort study of individuals at with intermediate cardiovascular risk (i.e., 1-5% 10-year risk of cardiovascular mortality events based on the SCORE tables). We included consecutive patients referred for coronary artery calcium score (CACS) appraisal at the national referral centre for cardiac CT, i.e., Institute of Radiology at the University Medical Centre in Ljubljana, Slovenia.

We collected baseline demographic and clinical data and venous blood samples for the measurement of lipid and coagulation parameters at baseline.

The study was approved by the National Ethics Committee of the Republic of Slovenia, No. 0120-161/2019/2019/4.

### 2.1. Baseline Data

At baseline, we collected data on age, sex, risk factors (smoking, hypertension, diabetes, and dyslipidemia), and comorbidities. Smoking use was defined as self-reported use of any nicotine product (including smoking and vaping) either currently (i.e., within the past 2 years) or in the past (>2 years prior to inclusion). Hypertension was defined as either self-reported diagnosis, usage of antihypertensive medication. or a blood pressure > 140/90 mmHg. Diabetes was defined either self-reported diagnosis or usage of antidiabetic medication. Dyslipidemia was defined as self-reported diagnosis; patients on lipid-lowering medication were however excluded from the study. Comorbidities included self-reported history of stroke, heart failure, myocardial infarction, or chronic obstructive lung disease. Physical activity (sedentarism or activity in minutes per week) was also self-reported.

### 2.2. Calcium Scoring

Computed tomographic assessment of CACS was performed using a 128-slice dual-source CT scanner (Somatom Drive; Siemens Medical Solutions, Forchheim Germany) with the following parameters: tube voltage 120 kV, automated anatomical tube current modulation (CARE Dose4D), reference tube current-time product of 80 mAs. and gantry rotation time 0.28 s. All images were reconstructed with a 3 mm slice thickness and an increment of 1.5 mm.

CACS of the lesions was calculated based on the weighted density score given to the highest attenuation value (HU) multiplied by the area of the calcified plaque, as previously described by Agatston et al. [23]. The density factor was assigned as 1 for lesions with maximal density 130-199 HU, 2 for lesions 200-299 HU, 3 for lesions 300-399 HU, and 4 for lesions > 400 HU. The score of all calcified lesions was summed up to give the total calcium score.

We defined the presence of CACS as a score of >0 Agatston units. For analysis purposes, the individuals in the present study were stratified in three groups: CACS 0, CACS 1-300, and CACS > 300.

### 2.3. Lipids, Antioxidant Status, and Hemostatic Biomarkers

The blood was collected on a single occasion from the antecubital vein according to the standard procedure and collected into two vacuum tubes: The first contained 0.11 mol/L sodium citrate, and the second contained clot activator and separator gel. The plasma was prepared by a 20-minute centrifugation at 2,500 × g and serum by a 20-minute centrifugation at 2,000 × g. Aliquots were prepared using plastic vials, snap frozen in liquid nitrogen, and stored at ≤-70°C.

HDL3 and sLDL concentrations were measured by the direct homogeneous method using HDL3-EX “SEIKEN” and sLDL-EX “SEIKEN” kits, respectively, on an RX Daytona+ automated chemistry analyzer (all Randox, Crumlin, United Kingdom). TAS was measured with the corresponding reagent kit on the same analyzer. In this assay, ABTS® (2,2′-azino-di-[3-ethylbenzthiazoline sulfonate]) is incubated with a peroxidase (metmyoglobin) and H_2_O_2_ to form the radical cation ABTS®+. This exhibits a relatively stable blue-green color which is measured at 600 nm. The antioxidants present in the sample suppress this color formation to an extent proportional to their concentration.

Parameters of the overall hemostasis potential assay (OHP, OCP, and OFP) were determined as described by He et al. [21]. Fibrin formation time curves were generated in microtitre plate wells, and plasma samples were tested in triplicate. For OHP measurement, microtitre wells contained 60 *μ*L plasma and OHP buffer (66 mmol/L Tris, 130 mmol/L NaCl, 17.0 mmol/L CaCl_2_, and pH 7.5) with 0.04 IU/mL bovine thrombin (Sigma, St. Luis, USA) and 348 ng/mL recombinant tissue-type plasminogen activator. OHP curves were generated from automated absorption measurements at 405 nm taken every minute for 40 min. OCP curves were obtained in an identical way, except that threaded buffer did not contain recombinant tissue-type plasminogen activator (Actilyse, Boehringer Ingelheim, Germany). Values for OCP and OHP were given by the areas under the relevant fibrin formation time curves calculated by summation of absorption values (Abs-sum). The OFP values in % were calculated as [(OCP − OHP)/OCP] × 100.

### 2.4. Statistical Methods

Baseline characteristics are expressed as mean ± standard deviation for normally distributed continuous variables, as median (interquartile range) for nonnormally distributed continuous variables, and as frequencies (%) for categorical variables. Between-group differences were assessed by *t*-test/ANOVA for normally distributed variables and by the Mann–Whitney *U* test/Kruskal-Wallis test for nonnormally distributed variables. Correlations were expressed by the Spearman correlation coefficient. Logistic regression models for prediction of a CACS zero were constructed for the multivariate analysis; results are expressed odds ratios with corresponding 95% confidence intervals (CI). A 2-tailed *p* < 0.05 was considered significant. Statistical analyses were carried out using SPSS Statistics version 23 (SPSS Inc, Chicago, USA).

## 3. Results

We included 160 patients; median age was 63 (interquartile range: 56-71) years, 52% were women, and median calcium score was 8 (IQR 0-173) Agatston units.

Across calcium score categories (0, 1-300, and >300 Agatston units, respectively), we detected significant differences in traditional risk factors (male sex, age, arterial hypertension, dyslipidemia, and diabetes), HDL3 levels, and TAS, but not in OHP, OCP, or OFP (Table 1). While male sex, age, arterial hypertension, dyslipidemia, and HDL3 levels were significantly different between patients without calcifications (calcium score 0) vs. with calcifications (calcium score 1-300 or >300 Agatston units), the differences in diabetes prevalence and TAS were only significant between patients with intermediate calcifications (1-300) versus severe calcifications (>300 Agatston units) on post hoc analysis.

HDL3 levels—but not LDL, sdLDL, triglyceride levels, TAS, or hemostatic potentials—were significantly (inversely) associated with presence of coronary calcifications; HDL3 retained statistical significance after multivariate adjustment for age, sex, arterial hypertension, dyslipidemia, diabetes, and smoking history (Table 2).

In further exploring possible association between selected biomarkers, HDL3 was significantly inversely associated with OCP (*r* = −0.232, *p* = 0.037) and sdLDL (*r* = −0.301, *p* = 0.009), but not with TAS (*r* = −0.072, *p* = 0.579), OHP (*r* = −0.162, *p* = 0.108), or OFP (*r* = −0.122, *p* = 0.177).

## 4. Discussion

In our study, HDL3 particles were inversely associated with coronary calcifications (i.e., calcium score) and the overall coagulation potential. Moreover, HDL3 emerged as an independent predictor of absence of coronary atherosclerotic vascular disease even after allowing for age, sex, and other traditional risk factors. Our findings suggest that HDL3 may be inversely associated with atherosclerotic vascular disease and a lower procoagulant (atherothrombotic) potential.

The role of HDL subtypes in atherosclerotic vascular disease is far from straightforward. Studies in patients with established atherosclerotic vascular disease suggested an unfavorable association between HDL3 levels and disease severity. In a case-control study of 312 individuals, Tian et al. detected higher HDL3 levels in patients with acute coronary syndromes as compared to patients with stable coronary artery disease and healthy controls [24]. Similarly, in patients with the metabolic syndrome, HDL3 levels are associated with dysmetabolic derangements irrespective of underlying coronary artery disease [25, 26]. Conversely, in apparently healthy populations, data seems to favor an inversed association between HDL3 and risk of atherosclerosis. Two large cohort studies in apparently healthy individuals have shown that HDL levels are inversely associated with incident coronary events at follow-up [27, 28]. HDL3 was (inversely) predictive of coronary calcifications in a large intermediate-risk cohort of individual population, although the association did not reach statistical significance when adjusting for age, traditional risk factors, and other conventional and novel lipid parameters [29]. Thus, in apparently healthy individuals—such as participants in our study—HDL3 seems to be associated with a decreased risk of atherosclerosis, whereas in patients with acute coronary syndromes or the metabolic syndrome, increased HDL3 levels seem to reflect cardiometabolic derangements, which are associated with increased cardiovascular risk.

Interestingly, as opposed to diagnosed (self-reported) dyslipidemia, none of the traditional lipid markers (LDL-C, HDL-C, and triglycerides) alone predicted coronary calcifications in our study, which is a likely result of selection bias. Patients referred for calcium scanning as per guidelines [30] fall in the intermediate-risk category (i.e., 1-5% predicted 10-year cardiovascular mortality rate in our study) with comparable lipid profiles (as depicted by low to moderate lipid derangements and relatively narrow value ranges in our study population). The clinical goal for subclinical atherosclerosis detection is lipid-lowering therapy initiation in patients below lipid thresholds; in this context, our findings suggest lipid subfraction measurement may improve prediction of subclinical coronary atherosclerosis in intermediate-risk populations.

Additionally, our results suggest that HDL3 levels are inversely associated with the concentration of sdLDL and OCP. On the one hand, the inverse association between HDL3 and sdLDL is an expected reflection of a dysmetabolic lipid profile [31]. On the other hand, the inverse association between HDL3 and OCP is a novel and interesting finding, as OCP is the part of the overall hemostatic potential suggesting a propensity towards a procoagulant state. In terms of atherothrombotic pathophysiology, this might suggest that high HDL 3 levels are associated with both, a lower burden of atherosclerotic vascular disease (i.e., lower CACS) and a lower potential for coagulation and thrombotic events (i.e., lower coagulation potential).

HDL levels are inversely associated with thromboembolic events, with several potential mechanisms explaining the effect of HDL on hemostasis [32]. HDL directly promotes anticoagulation (i.e., modulating the protein C pathway) [33] but may also affect hemostatic potentials indirectly—through modulation of the inflammation-coagulation cross talk (e.g., downregulation of E-selectin expression [34] and thrombin-induced endothelial cell tissue factor expression) [35], the improvement of endothelium-dependent hemostatic integrity (e.g., HDL inhibits endothelial cell apoptosis) [32], and the effects of HDL on platelet function [36]. Specific HDL3 versus HDL2 antithrombotic properties, however, remains more challenging to ascertain.

HDL structure and function derive from several proteins, which are differentially distributed between HDL3 and HDL2. Most of the HDL proteins have been traditionally associated with lipid metabolism (transport apoproteins, lipolytic enzymes, and transfer proteins) and atherosclerotic disease, whereas newer proteomic studies suggest additional anti-inflammatory and antithrombotic HDL protein functions [37]. Examples include the presence of fibrinogen, alpha-2 macroglobulin, platelet factor 4, and apolipoprotein H in HDL particles. The difference of functional protein distribution between HDL3 and HDL2 may partially explain the observed association between HDL3, CACS, and OCT in our study. Animal model studies also suggest that HDL3 specifically modulates inflammatory cytokine responses [38, 39] and the cyclooxygenase- (COX-) 2/prostacyclin pathways, thereby suggesting differential effects of HDL3 on inflammation and platelet function and hemostasis. Of note, HDL3 derangements, such as oxidation, may also play a role in the hemostatic effects of HDL3—i.e., oxydised HDL3, but not native HDL3, is associated with increased PAI-1 mRNA and antigen expression [40]. However, as we did not measure oxydised HDL3, such mechanistic explanations of our findings through HDL3 derangements remain purely speculative. We did, however, measure TAS, which was significantly associated with the level of coronary calcifications, but not with lipid parameters.

Our study has several limitations. Firstly, ours was a cross-sectional observational study; as such, it reports on possible association—but not causation—between dyslipidemia, hemostasis, and subclinical atherosclerosis. Also, it primarily informs on possible pathophysiological derangements in the process of atherothrombosis, but not on prognostic determination of relevant clinical events. Secondly, this was a single-centre study carried out at a tertiary national referral university hospital, and caution should be used when generalizing our results. Patient selection was contingent to appropriate guideline-directed use of CACS scanning, thereby resulting in a selected population at intermediate cardiovascular risk with mechanistic explanations limited to this subset of apparently healthy individuals. Thirdly, one-time measurements of lipid and hemostatic profiles may not represent levels of these parameters over a lifetime and do not address their relationship with potential outcomes.

In conclusion, in individuals at intermediate cardiovascular risk, HDL3 is associated with both, subclinical atherosclerosis (as determined by coronary calcifications) and overall hemostasis (as determined by *in vitro* analysis of coagulation and fibrinolitic potentials). Our findings are in line with studies reporting on an inverse relationship between HDL3 and atherosclerosis and provide one possible mechanistic explanation for the association between novel lipid biomarkers and coagulation derangements.

## Figures and Tables

**Table 1:** Baseline patient characteristics based on calcium score categories.

	Overall	CACS = 0	CACS 1-300	CACS > 300	*p* value
	*n* = 160	*n* = 65	*n* = 68	*n* = 27	
Age (years)	63 (56-71)	56 (49-63)^∗^	66 (58-72) ^∗^	71 (66-75)	<0.001 ^∗^
Sex (women)	84 (52.5%)	42 (64,6%) ^∗^	31 (45.6%) ^∗^	11 (40.7%)	0.024 ^∗^
Arterial hypertension	73 (45.6%)	23 (35.4%)^∗^	39 (57.4%) ^∗^	11 (40.7%)	<0.001 ^∗^
Dyslipidemia	54 (33.8%)	12 (18.5%) ^∗^	29 (42.6%) ^∗^	13 (48.1%)	0.002 ^∗^
Diabetes	16 (10.0%)	3 (4.6%)	6 (8.8%)^∗∗^	7 (25.9%)^∗∗^	0.005^∗∗^
Family history	87 (54.4%)	39 (60.0%)	31 (45.6%)	17 (63.0%)	0.105
Smoking			
No	86 (53.8%)	44 (67.7%)	32 (47.1%)	10 (37.0%)	0.057
Active	16 (10.0%)	4 (6.2%)	8 (11.8%)	4 (14.8%)	
Former	52 (32.5%)	15 (23.1%)	26 (38.2%)	11 (40.7%)	
Physical inactivity	101 (63.1%)	40 (61.5%)	45 (66.2%)	16 (59.3%)	0.859
HDL3	223 (196-257)	240 (221-269) ^∗^	226 (196-257) ^∗^	198 (174-218)	0.010 ^∗^
LDL	123 (97-147)	118 (89-147)	125 (104-147)	118 (97-124)	0.326
sdLDL	261 (212-369)	260 (234-376)	282 (206-365)	252 (163-322)	0.544
TAS	1.69 (1.54-1.74)	1.55 (1.49-1.66)	1.64 (1.56-1.73)^∗∗^	1.76 (1.59-1.88)^∗∗^	0.042^∗∗^
OHP	5.30 (3.80-7.84)	4.75 (2.91-8.44)	5.35 (4.28-7.55)	5.70 (4.96-7.48)	0.541
OCP	22.6 (18.9-22.0)	20.9 (17.5-25.1)	23-2 (19.4-27.1)	24.2 (21.7-28.7)	0.131
OFP	73.6 (66.6-80.0)	75.6 (63.1-80.1)	71.6 (68.6-77.7)	74.7 (22.8-81.0)	0.592

CACS: coronary artery calcium score; HDL3: high-density lipoprotein type 3; LDL; low-density lipoprotein; sdLDL: small dense low-density lipoprotein; TAS: total antioxidant status; OHP/OCP/OFP: overall hemostatic/coagulation/fibrinolytic potential. ^∗^ denotes significant difference at a <0.05 level between groups CACS 0 vs. CACS 1-300 on post hoc analysis; ^∗∗^ denotes significant difference at a <0.05 level between groups CACS 1-300 vs. CACS > 300 on post hoc analysis.

**Table 2:** Univariate and multivariate predictors of the presence of coronary clacifications.

	Univariate		Multivariate	
	OR (95% CI)	*p*	OR (95% CI)	*p*
Age (years)	1.134 (1.086-1.184)	<0.001	1.131 (1.048-1.221)	0.002
Sex (women)	2.187 (1.127-4.244)	0.021	1.782 (0.396-8.031)	0.452
Traditional risk factors			
Arterial hypertension	3.600 (1.829-7.087)	<0.001	0.482 (0.92-2.519)	0.387
Dyslipidemia	3.170 (1.496-6.718)	0.003	1.829 (0.449-7.452)	0.399
Diabetes	2.179 (0.677-7.021)	0.192	2.586 (0.185-36.214)	0.480
Smoking history	1.754 (1.202-2.560)	0.004	1.663 (0.759-3.647)	0.204
Lipids			
HDL3	0.981 (0.965-0.997)	0.019	0.980 (0.962-0.999)	0.034
LDL	1.001 (0.669-1.524)	0.962		
sdLDL	1.001 (0.998-1.003)	0.550		
Antioxidant status			
TAS	1.241 (0.086-17.853)	0.874		
Hemostatic biomarkers			
OHP	1.053 (0.897-1.236)	0.528		
OCP	1.043 (0.981-1.108)	0.181		
OFP	1.001 (0.972-1.031)	0.937		

OR: odds ratio of calcium score > 0 Agatston units; CI: confidence interval; HDL3: high-density lipoprotein type 3; LDL: low-density lipoprotein; sdLDL: small dense low-density lipoprotein; TAS: total antioxidant status; OHP/OCP/OFP: overall hemostatic/coagulation/fibrinolytic potential.

## Data Availability

The baseline demographic, clinical, and laboratory data used to support the findings of this study are available from the corresponding author upon request.
